# Proceedings of Societies

**Published:** 1871-08

**Authors:** 


### Proceedings of Societies.

### NORTH IOWA MEDICAL SOCIETY.

The North Iowa Medical Society held its 11th annual meeting at the office of Dr. Andros, McGregor, on Wednesday, June 7th.

Roll called, and found present — Drs. F. Andros, H. H. Clark, W. C. Lewis, J. S. Green, J. T. H. Scott, D. W. Chase, W. C. Earl and L. Brown, Jr.

Minutes of last meeting read and approved.

Drs. J. W. Curtis, of Decorah; A. B. Hanna, of Elkader; E. R. Zigler and S. E. Robinson, of West Union, were duly elected members of the Society.

Communications were received from Drs. N. H. Knowler, of Hesper, and Lewis L. Hazeltine, of New York City.

The following resolutions were adopted:

*Whereas*, in the conscientious discharge of duty, Dr. Lewis L. Hazeltine has removed from a sphere of usefulness which he has successfully exercised for the benefit of the public and honor of the profession; and

*Whereas*, from his first connection with this Society to its close, he was a faithful and efficient member; therefore,

*Resolved*, That we, the members of the North Iowa Medical Society, hereby express our regret at his loss, and unabated interest in his future welfare.

*Resolved*, That the Secretary be authorized to place his name upon the list of Honorary Members, and that a copy of our proceedings be at all times sent him.

The President, Dr. Andros, read his annual address, a well-written essay upon Anæsthetics, which brought out much discussion from several members; the discussion to be continued at the next meeting.

Dr. Curtis made a very lengthy report upon the value of the thermometer in the diagnosis and prognosis of disease.

Dr. H. H. Clark exhibited a fibrous tumor weighing three ounces, taken from the brain of a woman, who, although she had been sick for a long time, died so suddenly that foul play and poison were suspected.

There was a lengthy report upon the use and the effects of the hydrate f chloral. Dr. Brown had used it in two cases of puerperal convulsions, and one of partial insanity, caused by the poison of tobacco applied in the form of a poultice to a carbuncle; all three cases yielded promptly to it. Dr. Scott had used it in one, and Dr. Green in many cases of difficult labor, with the best success, causing sleep between pains, and relaxation, more than any other drug. Dr. Chase, of Elkader, reported a case, a patient of his, a physician who, upon his own responsibility, took over one ounce to a dose without producing any deleterious effects, only thirty hours profound sleep.

Dr. Scott reported an interesting case of strangulated scrotal hernia in a young man whom he and Dr. Andros operated on, and found a large portion of the omentum mortified which they cut away, and ligated the stump, fastened one end of the ligature external to the wound; in a few days it became loose, and was drawn up into the abdomen. Several weeks after, an abscess appeared in the umbilical region, which discharged a few ounces of pus and the ligature. The patient then made a rapid recovery.

Many interesting cases were reported by different members, and opinions expressed.

The Constitution was changed so as to admit Howard county to the territory of the Society.

On motion of Dr. S. E. Robinson, the President was directed to appoint a Committee of one to report upon Surgery, Obstetrics, Practice, and new remedies, and one or two other subjects, to report at the next annual meeting.

The following officers were elected for the ensuing year:

W. C. Lewis, of Clermont, President; Luther Brown, Jr., Postville, Vice President; J. S. Green, Postville, Secretary; H. H. Clark, McGregor, Treasurer; Drs. J. S. Green, J. T. H. Scott and H. H. Clark, Censors.

Drs. F. Andros and J. W. Curtis, were appointed Delegates to the Amer. Med. Ass'n; and Drs. F. Andros, J. W. Curtis and H. H. Clark, Essayists.

The Secretary was authorized to make credentials to any member who would attend the State Medical Society.

Adjourned, to hold the next annual meeting at Postville, on the first Wednesday of June, 1872; also a semi-annual meeting at Decorah, on Wednesday, June 25, 1871.

All regular physicians in good standing are requested to attend.

J. S GREEN, *Secretary.*

---

### DeWITT COUNTY MEDICAL SOCIETY.

The DeWitt County Medical Society met in annual session, at the office of Dr. C. Goodbrake, in Clinton, on Tuesday, the 2d day of May, A. D. 1871, the President, Dr. W. G. Cochran, in the chair.

Present—Drs. W. G. Cochran, J. H. Tyler, J. A. Edmiston, W. W. Adams, Z. H. Madden, B. S. Lewis, C. Goodbrake, J. Wright, and T. Kelley.

The minutes of the last regular meeting were read and approved.

The election of officers being in order, the following named gentlemen were elected for the ensuing year.

President—Dr. C. Goodbrake; Vice President—Dr. J. H. Tyler; Secretary

—Dr. Thomas Kelley; Treasurer—Dr. J. A. Edmiston; Censors—Drs. J. Wright, Z. H. Madden, and Wm. W. Adams.

Delegates to the State Medical Society, (which meets in Peoria, on the third Tuesday in May, 1871,) Drs. C. Goodbrake, Z. H. Madden, J. H. Tyler, and J. A. Edmiston.

On motion, the election of delegates to the American Medical Association was postponed until the regular meeting in January, 1872.

On motion, the Society adjourned, to meet again at one o'clock P. M.

AFTERNOON SESSION.

Society met pursuant to adjournment.

Present—Drs. C. Goodbrake, J. H. Tyler, T. K. Edmiston, J. A. Edmiston, B. S. Lewis, J. Wright, W. G. Cochran, Z. H. Madden, T. W. Davis, J. H. Potter, N. Patterson, T. Kelley, and D. W. Edmiston. Dr. Laughlin, ot Bloomington, honorary member of this Society, was also present.

By the request of the retiring President, he was excused from delivering his valedictory address until the next regular meeting.

Dr. Wm. W. Adams read a very able essay on Typhoid Fever.

On motion of Dr. T. K. Edmiston, Dr. W. W. Adams was requested to furnish the Society a copy of his essay for publication in the *Chicago Medical Journal.*

Dr. D. W. Edmiston read a very interesting essay on Diseases of the Air Passages.

On motion, Drs. W. W. Adams and D. W. Edmiston were continued as essayists for the next regular meeting.

On motion, the chair appointed Drs. T. K. Edmiston, W. W. Adams and Thomas Kelley, a committee to draft resolutions, expressive of the sense of this Society in regard to the death of the late Dr. Harrison Noble, honorary member of this Society.

On motion of Dr. T. K. Edmiston, the President, Dr. C. Goodbrake, was added to the above committee.

On motion of Dr. W. G. Cochran, Dr. J. A. Edmiston was requested to furnish the Society an essay on Materia Medica and Pharmacy.

On motion, the Chair appointed Drs. Wm. G. Cochran, J. Wright, and T. Kelley, a committee to correspond with Medical Societies of the adjoining counties in regard to forming a District Medical Society.

Dr. Laughlin reported a case of General Paralysis of much interest to the Society.

On motion, Cholera Infantum was made the subject of discussion for the next regular meeting.

On motion, the Secretary was requested to furnish the editors of both of the Clinton papers, and the *Chicago Medical Journal*, with copies of the proceedings for publication.

On motion, the Society adjourned, to meet in Marion on the second Tuesday in July next.

THOMAS KELLEY, *Sec.*

